# Early-stage HER2-positive breast cancer in neurofibromatosis type 1: a case report with literature review

**DOI:** 10.3389/fonc.2026.1930403

**Published:** 2026-09-22

**Authors:** Fulan Yang, Sujuan Dai, Liyin Huang, Zhaoqi Liu, Juan Wang, Xun Xi

**Affiliations:** 1 Department of Breast Surgery, Ganzhou People’s Hospital, Ganzhou, Jiangxi, China; 2 Department of Pathology, Ganzhou People’s Hospital, Ganzhou, Jiangxi, China; 3 Department of Ultrasound, Ganzhou People’s Hospital, Ganzhou, Jiangxi, China; 4 Department of Radiology, Ganzhou People’s Hospital, Ganzhou, Jiangxi, China

**Keywords:** breast neoplasms, case report, early detection of cancer, mastectomy, neurofibromatosis type 1

## Abstract

Neurofibromatosis type 1 (NF1) is an autosomal dominant disorder associated with an increased risk of malignancies, particularly breast cancer at a younger age. We report the case of a 48-year-old woman with clinically diagnosed NF1 who presented with a left breast mass detected during routine screening. Physical examination revealed numerous café-au-lait macules and cutaneous neurofibromas distributed over the trunk and extremities. Imaging findings were classified as BI-RADS 4B–4C. Following intraoperative confirmation of malignancy, total mastectomy and sentinel lymph node biopsy were performed. Histopathological examination demonstrated HER2-positive invasive ductal carcinoma with associated ductal carcinoma *in situ* (pT1cN0M0, Stage IA), while all sentinel lymph nodes were negative for metastasis. The patient subsequently received adjuvant chemotherapy combined with trastuzumab. No recurrence or distant metastasis was observed during 21 months of follow-up. This case highlights the importance of early screening, individualized treatment, multidisciplinary management, and regular surveillance to optimize outcomes in patients with NF1-associated breast cancer.

## Introduction

Neurofibromatosis type 1 (NF1) is a common autosomal dominant disorder caused by pathogenic variants in the *NF1* gene located on chromosome 17q11.2, which encodes the tumor suppressor protein neurofibromin (1, 2). NF1 affects approximately 1 in 3,000 individuals worldwide, with 30%–50% of cases arising from *de novo* mutations (3). Typical manifestations include café-au-lait macules, neurofibromas, freckling, Lisch nodules, and skeletal abnormalities (1). NF1 is a cancer predisposition syndrome characterized not only by susceptibility to cognitive impairment and vascular abnormalities but also by an elevated risk of a broad range of malignancies, including malignant peripheral nerve sheath tumors, gliomas, pheochromocytomas, gastrointestinal stromal tumors, and breast cancer (1, 4).

Recent studies have demonstrated that women with NF1, particularly those younger than 50 years of age, have an approximately fivefold increased risk of developing breast cancer compared with the general population, with cumulative risk estimated to reach approximately 8–13% by age 50 (4). NF1-associated breast cancer is frequently characterized by hormone receptor negativity, HER2 overexpression, and high histological grade, features that are generally associated with a poorer prognosis (5, 6). Evidence regarding optimal screening, treatment, and long-term follow-up remains limited (5, 6). Herein, we report a rare case of early-stage breast cancer in a patient with NF1 and review the current literature to provide further insights into its clinicopathological characteristics and management.

## Case report

A 48-year-old woman with a history of NF1, diagnosed at 15 years of age based on characteristic clinical manifestations fulfilling NF1 diagnostic criteria, was found to have a hypoechoic nodule in the left breast during routine breast ultrasonographic screening on June, 2024, and was subsequently referred to the Department of Breast Surgery at Ganzhou People’s Hospital. She had no family history of NF1 or related manifestations among her grandparents, parents, siblings, or children (a 19-year-old son and a 16-year-old daughter), suggesting a sporadic presentation. Her children have undergone clinical evaluation for NF1, but formal genetic testing has not been performed. There is no known family history of breast cancer or other malignancies among first- or second-degree relatives. She had no history of other NF1-associated complications, including optic pathway glioma, skeletal abnormalities, vascular complications, malignant peripheral nerve sheath tumors, or other NF1-associated malignancies. Clinical and radiological evaluations revealed no evidence of plexiform neurofibromas. Physical examination revealed a firm, poorly defined mass measuring approximately 2.0 × 1.0 cm in the upper outer quadrant of the left breast. Multiple café-au-lait macules and numerous cutaneous neurofibromas of varying sizes were observed diffusely on the trunk, extremities, and breasts, including relatively large nodules involving the nipple–areola complex (**Figure 1**). The patient reported no breast pain, nipple discharge, or discomfort associated with the cutaneous neurofibromas.

**Figure 1:**
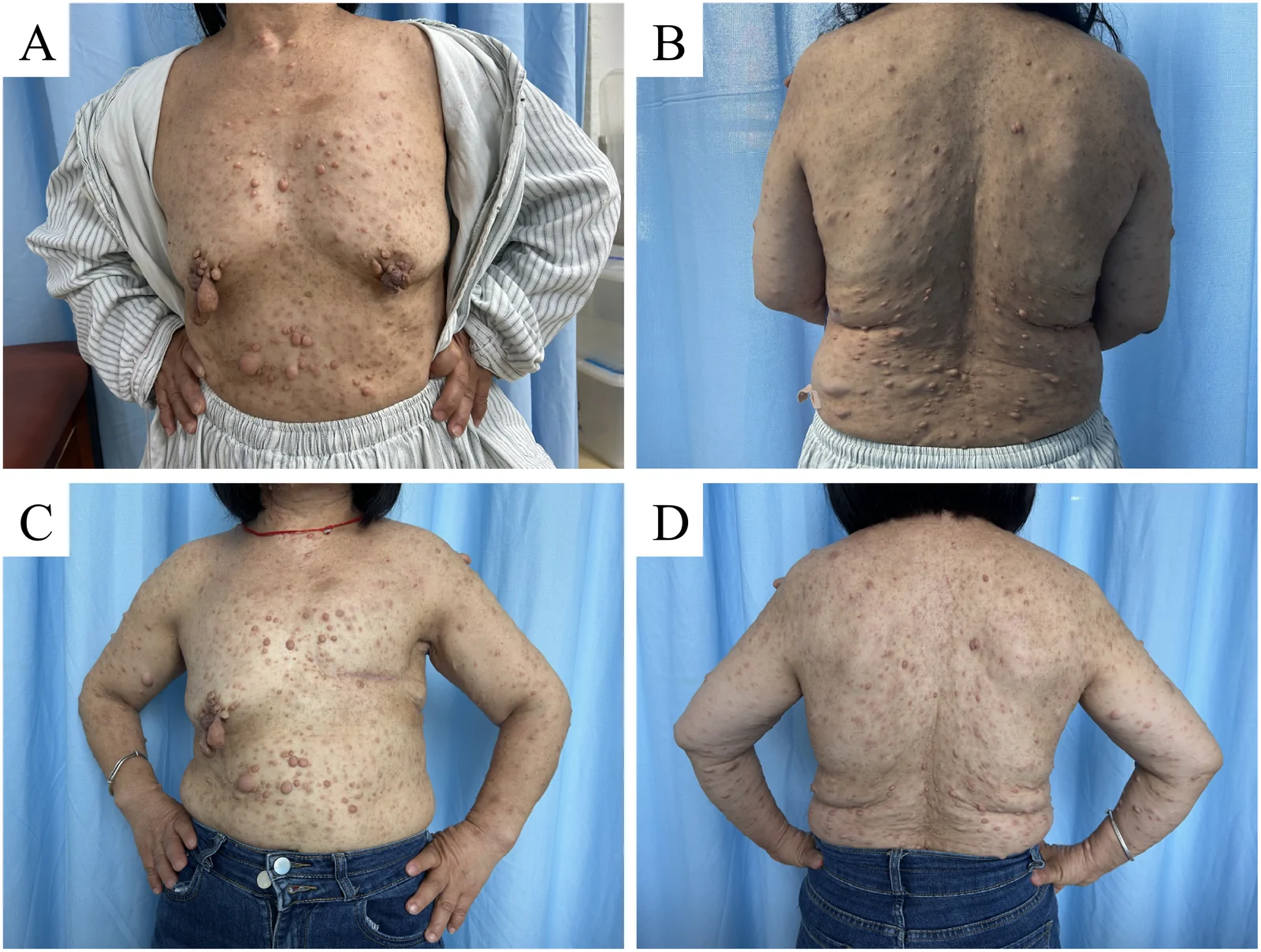
Clinical photographs obtained before and after breast surgery. **(A)** Preoperative anterior view; **(B)** preoperative posterior view; **(C)** postoperative anterior view; and **(D)** postoperative posterior view. The patient was diagnosed with neurofibromatosis type 1 and invasive ductal carcinoma of the breast. Multiple café-au-lait macules and cutaneous neurofibromas of varying sizes are visible on the skin.

Breast ultrasonography demonstrated an irregular hypoechoic lesion measuring 2.2 × 1.2 cm with internal vascularity and was categorized as BI-RADS 4B (**Figure 2A**). Breast magnetic resonance imaging (MRI) revealed an irregular mass measuring 1.2 × 2.6 × 1.8 cm with heterogeneous enhancement and a washout-type time–signal intensity curve, corresponding to BI-RADS 4C (**Figure 2B**). Mammography demonstrated multiple fine polymorphic calcifications distributed segmentally and was also categorized as BI-RADS 4B (**Figure 2C**). Preoperative staging evaluation included breast ultrasonography, mammography, and breast MRI for local assessment, as well as contrast-enhanced CT of the chest and abdomen and brain MRI for metastatic evaluation. No evidence of regional lymph node involvement or distant metastasis was identified on preoperative evaluation. The patient declined preoperative core needle biopsy. In view of the coexistence of NF1-related neurofibromas, benign breast lesions and primary breast malignancy were considered in the differential diagnosis; however, the newly developed irregular lesion with suspicious imaging features warranted pathological evaluation. Because the HER2 status was not established prior to surgery, neoadjuvant HER2-targeted therapy could not be administered. Furthermore, preoperative imaging suggested a relatively small and localized lesion without evidence of axillary or distant metastasis, which supported proceeding directly to surgical excision. Given the suspicious imaging findings (BI-RADS 4B–4C), the relatively localized lesion, and the need for definitive pathological diagnosis, excisional biopsy was performed. Intraoperative frozen-section analysis revealed invasive carcinoma, prompting immediate left total mastectomy and sentinel lymph node biopsy. Examination of three sentinel lymph nodes demonstrated no evidence of metastatic involvement.

**Figure 2:**
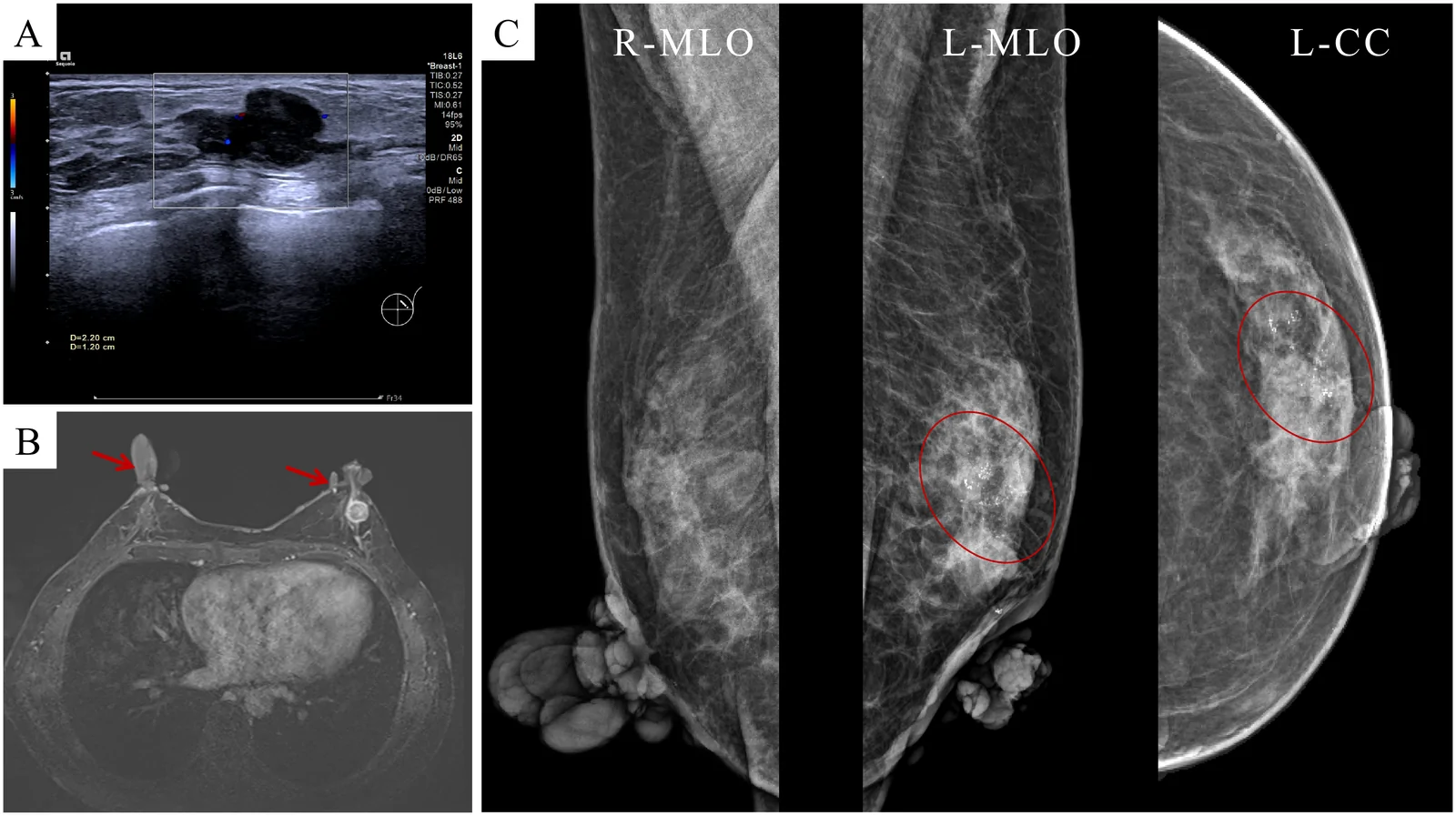
Imaging findings. **(A)** Breast ultrasonography showing a poorly defined hypoechoic mass in the left breast with internal vascularity and punctate calcifications. The lesion measures approximately 2.2 × 1.2 cm. **(B)** Breast magnetic resonance imaging demonstrating marked heterogeneous enhancement of the left breast lesion. Multiple small nodular lesions (red arrows) are also visible in the bilateral nipple–areolar complexes, consistent with neurofibromatosis. **(C)** Mammographic craniocaudal and mediolateral oblique views of the left breast showing multiple fine pleomorphic calcifications (red ovals). Multiple nodular areas of increased density are also present in the nipple–areolar region, consistent with neurofibromatosis.

Histopathological analysis confirmed invasive ductal carcinoma (IDC) measuring 1.5 cm in greatest dimension, accompanied by well-differentiated ductal carcinoma *in situ*. Surgical margins were negative, and no lymphovascular or perineural invasion was identified (**Figures 3A–H**). Subcutaneous nodules excised during the procedure were histologically confirmed as neurofibromas (**Figures 3I–L**). Immunohistochemical analysis demonstrated estrogen receptor (ER)-negative, progesterone receptor (PR)-negative, and HER2-positive (3+) status, with a Ki-67 proliferation index of 35%. The tumor was negative for CK5/6 and exhibited preserved E-cadherin expression. The final diagnosis was HER2-overexpressing IDC, staged as pT1cN0M0 (Stage IA), with coexistent NF1.

**Figure 3:**
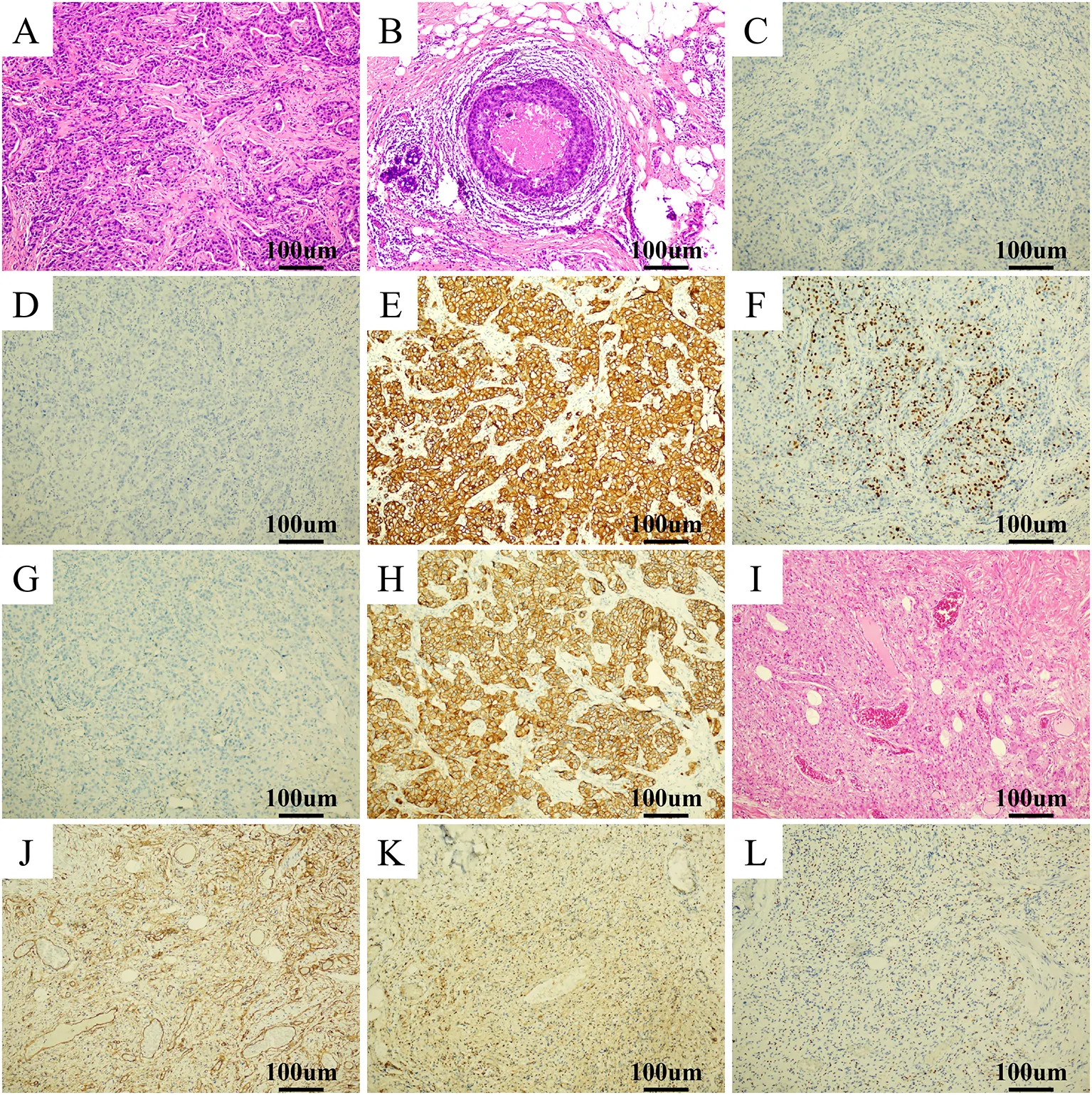
Histopathological and immunohistochemical findings of breast carcinoma and cutaneous neurofibroma specimens. **(A–B)** Hematoxylin and eosin (H&E) staining demonstrating invasive ductal carcinoma **(A)** and high-grade ductal carcinoma *in situ* **(B)**. **(C–H)** Immunohistochemical staining of invasive ductal carcinoma showing: **(C)** estrogen receptor, negative; **(D)** progesterone receptor, negative; **(E)** HER2, score 3+; **(F)** Ki-67 labeling index, 35%; **(G)** cytokeratin 5/6 (CK5/6), negative; and **(H)** E-cadherin, membranous positive. **(I)** H&E staining of a cutaneous neurofibroma specimen. **(J–L)** Immunohistochemical staining of neurofibroma showing: **(J)** CD34, positive; **(K)** S100, positive; and **(L)** SOX10, positive. Scale bar = 100 μm. Original magnification, x100.

Adjuvant treatment consisted of four cycles of epirubicin plus cyclophosphamide followed by four cycles of docetaxel, together with 1 year of trastuzumab therapy. The patient completed the planned adjuvant treatment regimen without treatment interruption. The treatment was well tolerated, with no serious treatment-related adverse events or unexpected complications observed. At the most recent follow-up (21 months after surgery), regular physical examinations and imaging evaluations revealed no evidence of local recurrence or distant metastasis. The patient remains under scheduled follow-up. A summary of the diagnostic, therapeutic, and follow-up timeline is provided in **Figure 4**.

**Figure 4:**
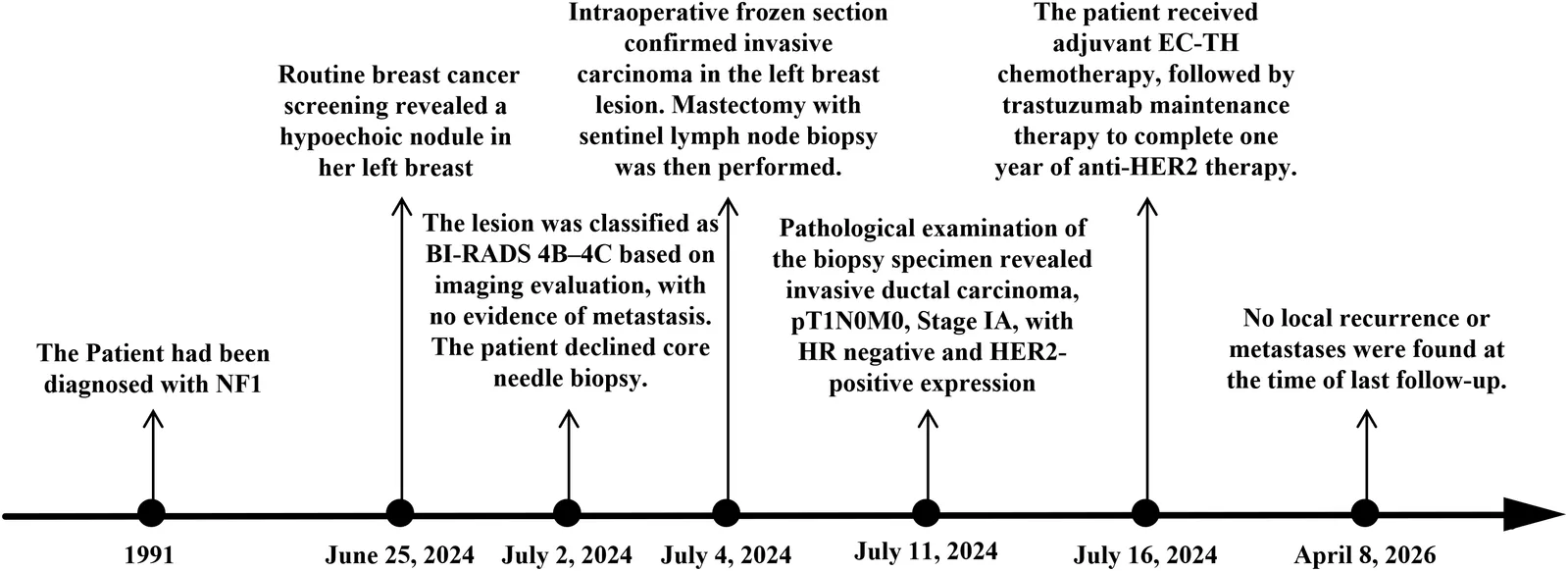
Timeline of diagnosis, treatment, and follow-up. NF1, neurofibromatosis type 1; HER2, human epidermal growth factor receptor 2; EC, epirubicin plus cyclophosphamide; T, docetaxel; H, trastuzumab. Epirubicin plus cyclophosphamide was administered every 3 weeks for four cycles, followed by docetaxel combined with trastuzumab every 3 weeks for four cycles. Trastuzumab was subsequently continued to complete 1 year of anti-HER2 therapy.

## Discussion

This case involved HER2-overexpressing IDC in a 48-year-old patient with NF1. The patient presented with characteristic cutaneous features of NF1, including multiple café-au-lait macules and numerous cutaneous neurofibromas distributed throughout the body. A suspicious lesion was identified in the left breast during the patient’s initial routine breast ultrasound screening, leading to a final diagnosis of IDC. Although cutaneous manifestations do not necessarily prevent breast examination or imaging evaluation, extensive visible neurofibromas may contribute to delayed healthcare-seeking behavior and reduced participation in routine surveillance. The patient achieved a favorable prognosis as a result of active treatment. This case highlights the importance of proactive breast surveillance and timely intervention in patients with NF1, and further demonstrates the effectiveness of standard breast cancer treatment in this population (7).

Germline NF1 genetic analysis was not pursued in this patient. The revised 2021 diagnostic criteria for NF1 require the presence of at least two characteristic clinical, radiological, genetic, or familial features (8). Because NF1 remains primarily a clinical diagnosis, genetic testing is not mandatory for diagnosis (8). Molecular testing is generally reserved for atypical presentations, suspected mosaic NF1, family risk assessment, or research (2, 9). In the present case, the diagnosis of NF1 was established based on characteristic clinical findings, including multiple café-au-lait macules and cutaneous neurofibromas. An additional reason for not pursuing genetic testing was that NF1 genetic analysis was not covered under the patient’s health insurance plan. Tumor genomic sequencing was not performed, as the results would not have influenced the adjuvant treatment strategy for this early-stage HER2-positive breast cancer.

Neurofibromin functions as a tumor suppressor by acting as a Ras GTPase-activating protein (GAP), which negatively regulates Ras signaling and maintains cellular homeostasis; its loss results in persistent activation of downstream signaling pathways, including Ras/MAPK, PI3K/AKT/mTOR, and cAMP-related pathways, thereby promoting abnormal cellular proliferation and tumorigenesis (1, 10). These alterations contribute not only to neurofibroma formation but also to increased susceptibility to various malignancies (1, 6), establishing NF1 as a cancer predisposition syndrome associated with elevated lifetime risks of malignant peripheral nerve sheath tumors, gliomas, pheochromocytomas, gastrointestinal stromal tumors, and breast cancer (1). This heightened susceptibility is largely attributable to the loss of neurofibromin-mediated tumor suppression and the consequent dysregulation of multiple oncogenic signaling networks (1, 10).

Wang et al. first demonstrated that NF1-associated breast cancers exhibit distinct molecular characteristics, including HER2 overexpression, hormone receptor negativity, and activation of Ras/MAPK-related signaling pathways, which may contribute to their aggressive biological behavior (11–13). Several genomic investigations have identified NF1 alterations as recurrent molecular events in breast cancer, particularly in hormone receptor-positive and HER2-enriched subtypes (11, 13). Loss of NF1 may induce resistance to endocrine therapy through activation of alternative growth signaling pathways, while cooperative effects between NF1 loss and HER2 amplification may further enhance tumor proliferation and survival signaling; interactions with BRCA1, ERBB2, and TP53 may also promote tumorigenesis (11, 13). These molecular characteristics may partly explain the relatively aggressive clinical course observed in NF1-associated breast cancer. Notably, previous studies have suggested that NF1-associated breast cancer tends to occur at a younger age and presents with unfavorable pathological features—such as larger tumor size, higher histological grade, and increased HER2 expression—compared with sporadic breast cancer; however, considerable heterogeneity exists among reported cases (5, 6, 14). In the present patient, despite the presence of HER2-positive disease, early detection through imaging surveillance and the absence of lymph node involvement likely contributed to a favorable prognosis, which is consistent with prior reports of NF1-associated breast cancer presenting as HER2-positive IDC with multiple cutaneous neurofibromas.

In addition to breast cancer, individuals with NF1 are at increased risk for malignant peripheral nerve sheath tumors, skeletal abnormalities, cognitive impairment, and vascular disorders (1); therefore, regular surveillance and multidisciplinary management are essential for early detection and improved overall outcomes. The favorable prognosis observed in this patient suggests that, even in the context of the aggressive biological features characteristic of NF1-associated breast cancer, early detection and appropriate guideline-based treatment confer potential benefits (7).

Management of breast cancer in patients with NF1 requires individualized treatment planning. Although breast-conserving surgery followed by radiotherapy remains a standard treatment option for many patients with early-stage breast cancer (7), its safety profile in NF1 patients remains incompletely defined. To date, no studies have directly demonstrated an increased risk of radiation-induced breast malignancy specifically in NF1 patients. Mechanistic studies have suggested that neurofibromin participates in DNA damage repair, and histopathological observations have raised concerns regarding radiation-induced transformation of neurofibromas into MPNST (16, 17). A systematic review demonstrated that radiation-induced MPNSTs carry a particularly poor prognosis (median overall survival: 11 months), with complete surgical resection remaining the mainstay of treatment (18). However, controversial conclusions exist regarding the radiation risk on PN or MPNST alone, and clinical evidence remains inconclusive. A recent cohort study of 27 NF1 breast cancer patients treated with adjuvant radiotherapy represents the largest clinical series to date addressing this question (19). Nevertheless, given the retrospective nature of available evidence, the risk–benefit ratio of radiotherapy in NF1-associated breast cancer ultimately requires future well-designed cohort studies and controlled trials. Therefore, while the biological plausibility of heightened radiation sensitivity in the context of NF1 deficiency warrants continued investigation, definitive clinical guidance cannot be established based on current limited evidence, and clinical decisions should be made with individualized consideration. In the present case, several factors further tilted the balance away from breast-conserving surgery. Notably, the presence of scattered multifocal microcalcifications on mammography represented a relative contraindication to breast-conserving surgery, raising concerns regarding the extent of local disease and the potential challenges in achieving optimal local control with a conservative approach (7). In addition, the patient herself did not desire breast-conserving surgery and preferred a definitive surgical procedure. Following multidisciplinary discussion and careful consideration of the imaging findings, disease extent, and patient preference, total mastectomy was therefore performed, yielding negative surgical margins and excellent local control. Moreover, although HER2-targeted neoadjuvant therapy represents an important option for selected patients with HER2-positive breast cancer (7), it was not pursued in this case because the HER2-positive status was not established prior to surgery, and preoperative imaging had suggested a relatively small and localized lesion without evidence of axillary or distant metastasis.

Postoperatively, based on the final pathological findings (pT1cN0M0, stage IA, HER2-positive IDC), adjuvant chemotherapy combined with trastuzumab was administered, in accordance with current treatment principles for early-stage HER2-positive breast cancer (7). Given the tumor’s higher-risk biological profile—including hormone receptor negativity, HER2 overexpression, and an elevated Ki-67 proliferation index (35%)—along with the aggressive characteristics reported in NF1-associated breast cancer, an intensive chemotherapy regimen was selected after multidisciplinary deliberation. Overall, this case supports the application of standard systemic therapy in NF1 patients, while underscoring the importance of individualized surgical decision-making and the need for long-term follow-up in this population.

Epidemiological studies have demonstrated that women with NF1 have a substantially increased risk of developing breast cancer before the age of 50 years and may experience poorer overall survival compared with the general population (4, 15). Accordingly, recent cohort studies have recommended initiating annual breast surveillance (mammography or ultrasonography, with or without MRI) starting at age 30–40 years in NF1 patients; however, optimal screening strategies remain to be fully defined, as current evidence is mainly derived from retrospective cohorts and limited population-based studies (15, 20, 21). Further studies are needed to characterize NF1-associated breast cancer and optimize screening and treatment.

As NF1 is an autosomal dominant genetic disorder, genetic counseling plays an important role in patient management. Although this patient had no family history of NF1 or malignancies, counseling regarding the potential risk of transmission to offspring, the implications of NF1-related tumor susceptibility, and appropriate surveillance strategies should be considered. Formal genetic counseling was not provided to this patient, largely due to the clinical focus on oncological management at the time of treatment and the patient’s personal preference. Although germline genetic testing was not pursued in this case due to insurance constraints, it is worth noting that such testing, if available, could provide valuable information for cascade screening of at-risk family members. Identification of a pathogenic NF1 variant would enable targeted surveillance and early intervention for NF1-associated manifestations, including breast cancer, in first-degree relatives. Multidisciplinary management should incorporate not only oncological treatment but also genetic education and psychosocial support to address the broader impact of this hereditary tumor syndrome. In addition to oncological outcomes, the potential psychosocial impact of NF1-related visible cutaneous manifestations and breast cancer should also be considered. In this patient, the presence of extensive cutaneous neurofibromas may have contributed to psychological burden and delayed healthcare-seeking behavior; however, formal psychological assessment and standardized patient-reported outcome measures were not performed, representing a limitation of this report. Moreover, as this study describes a single case, the clinical characteristics, treatment response, and outcomes observed in this patient cannot be generalized to all individuals with NF1-associated breast cancer. Future prospective studies incorporating validated patient-reported outcomes and psychological evaluations are warranted to better understand the psychosocial impact and support comprehensive, patient-centered management of NF1-associated breast cancer.

## Conclusion

This case highlights the increased risk and distinctive molecular features of NF1-associated breast cancer, particularly HER2 overexpression. Early detection through imaging surveillance enabled timely treatment. Multidisciplinary management resulted in favorable outcomes without recurrence during follow-up. These findings underscore the importance of proactive screening, individualized management, and long-term surveillance in patients with NF1.

## Data Availability

The original contributions presented in the study are included in the article/supplementary material. Further inquiries can be directed to the corresponding author.
